# Evaluation of implants in smoking and non-smoking patients with peri-implant disease risk analysis and esthetic scores: an observational study

**DOI:** 10.1186/s12903-023-03696-3

**Published:** 2023-11-25

**Authors:** Tuğba Şahin

**Affiliations:** Dentistry Faculty, Division of Periodontology, Bolu Abant İzzet Baysal University, Bolu, Türkiye

**Keywords:** Dental implant, Implant Disease risk assessment, Keratinized tissue, Pink esthetic score

## Abstract

**Background:**

This study examined how smoking affects esthetics, peri-implant health, gingiva around the implant, and implant disease risk assessment in patients with implants.

**Methods:**

The study included two hundred ninety-eight implants of systemically healthy patients aged between 38 and 62 who applied to the Periodontology Clinic and whose functionally prosthesis-loaded implants had been at least six months and at most five years old. Implants of patients with bruxism were not included in the study. Implants are divided into two according to the patient’s smoking. Vestibule depth around the implant, keratinized gingival thickness and width, gingival recession, bleeding on probing, pocket depth, and gingival index by a sole clinician. The pink esthetic score, peri-implant disease risk assessment, and implant health scale were also examined to measure implant esthetics and success.

**Results:**

There was a statistically significant difference in the implant disease risk assesment scores for the examined implants of smokers and nonsmokers (p < 0.05). People who had peri-implantitis had higher implant disease risk assesment score levels. The dental implant health scale revealed a statistically significant difference (p < 0.05) in the likelihood of implant disease. According to the dental implant health scale, dental implants were 100% successful for non-smokers. There was a significant difference in the keratinized gingiva width between smokers and nonsmokers (p < 0.05). The results of the study showed that nonsmokers had a wider keratinized gingiva.

**Conclusions:**

Research has demonstrated that the act of smoking has the potential to jeopardize the long-term survival of dental implants and the surrounding peri-implant tissues. The results of this study indicate that it would be advisable for dentists to provide guidance to their patients on smoking cessation and to monitor any alterations in behavior closely. Furthermore, it would be advantageous for dental professionals to elucidate the impact of smoking on the susceptibility of smokers to peri-implant disease.

## Background

The oral environment and its ecology, gingival tissues, vasculature, inflammatory and immunological responses, and the homeostasis and capacity for healing of periodontal connective tissues are all impacted by cigarette smoking [1]. Dental implant therapy is one of the best options for restoring lost teeth [2]. Implant care over the long term may be threatened by pathological disorders, including peri-implant mucositis and peri-implantitis [3]. Potential risk factors can increase the susceptibility to peri-implant diseases at both the implant and the patient levels [4]. The evidence linking diabetes mellitus and cigarette smoking to peri-implantitis is still unclear, and these moderating variables are considered potential risk indicators or emerging risk factors [5]. There is substantial evidence that poor oral hygiene, history of periodontitis, and cigarette smoking are associated with peri-implant diseases [6]. Smoking significantly reduces neutrophil transmigration across the periodontal microvasculature and causes systemic neutrophilia. It has been shown that smoking inhibits chemokinesis, chemotaxis, phagocytosis, and neutrophil cell spreading. Neutrophils’ protease release may be crucial in tissue deterioration [1]. Nicotine causes an increase in alveolar bone loss by promoting the formation and proliferation of osteoclasts [7]. In addition, different protocols have been developed against traditional methods to improve oral hygiene habits, which is another crucial risk factor for peri-implant diseases [8].

The success or failure of an implant is not just determined by periodontal indicators [9]. These clinical indices must be connected to additional elements, such as exudate or prosthesis overloading [10]. Compared with implant success or survival, implant failure is more straightforward to explain and can be caused by several variables. Implant removal is warranted in cases of pain, vertical movement, or uncontrollably progressing bone loss [11]. Plaque assessment, mucosal conditions, peri-implant probing depth, peri-implant sulcus fluid analysis, suppuration, implant mobility, discomfort, resonance frequency analysis, and radiographic evaluation are among the parameters that may be used to evaluate peri-implant health and the severity of peri-implant disease [12]. The thickness of the tissue around the dental implant is crucial, along with the other factors [13]. It is suggested that 2 mm of keratinized gingiva (corresponding to 1 mm attached gingiva in the material used in this study) is adequate for maintaining gingival health [14].

The pink esthetics score (PES) makes it possible to evaluate the esthetic outcomes of different surgical and prosthetic implant techniques more objectively, both in the short and long term [15]. Furthermore, it generates robust intraexaminer agreement and consistently evaluates the soft tissue around individual implant restorations [16].

The demand for a risk assessment tool that can forecast the development of peri-implantitis and is comparable to the one created for periodontal diseases has grown along with interest in the etiology and pathophysiology of peri-implant infections. The peri-Implant disease risk assessment (IDRA) graphic was designed to reduce the consequences of peri-implant tissue damage [17]. The IDRA algorithm is a valuable tool for determining whether a patient is at moderate or high risk for developing peri-implantitis [18]. Based on the data collected for this study, this risk assessment tool can be used to diagnose peri-implant disease early on. Determining particular risk variables may increase the likelihood of an implant surviving [19]. This study aimed to examine the effects of smoking on esthetics, peri-implant health status, and the condition of the gingiva around the implant, along with the peri-implant disease risk assessment diagram in patients with implants.

## Methods

### Study design and participants

This research complied with the World Medical Association Declaration of Helsinki on medical research [20]. The Ethical Review Board approved the study of the Bolu Abant İzzet Baysal University, Bolu (2022/160). This clinical study was registered at ClinicalTrials.gov (NCT05823038).

This study was conducted at a single center (Bolu Abant İzzet Baysal University, Dentistry Faculty, Division of Periodontology, Bolu, Turkey). A clinician documented the medical histories of individuals receiving implants (T.Ş.). Individuals with uncontrolled diabetes and bruxism were excluded from the trial, leaving 298 implants whose functional prosthetic loading had not occurred for at least six months and at most five years old. The patients who came to have their implants examined and did not undergo guided bone regeneration were included in the study. Patients mainly were split into two groups: nonsmokers and smokers. Those who smoked 100 cigarettes or more in their life and still smoked were grouped as smokers, and those who smoked less than 100 cigarettes in their life and did not currently smoke were grouped as non-smokers [21]. The concept of the suggested treatment, in addition to its advantages and disadvantages, was explained to the participants, who then signed consent forms. It turned out that the patients did not utilize dental hygiene products explicitly designed for implants, nor did they have regular implant checkups.

Plaque, gingival index, bleeding on probing, pocket depth, gingival recession, clinical attachment loss, keratinized thickness, keratinized width, vestibule depth, PES, dental implant health scale, and IDRA were all systematically assessed in each implant in patient. The pink aesthetic score examined papilla, soft tissue margin level, soft tissue contour, alveolar process, soft tissue color, and texture. The pink esthetic score is assessed on a scale ranging from 0 to 10 [16]. Four groups on the implant health scale define the clinical conditions of success, satisfactory survival, compromised survival, or failure. While making these evaluations, pain, mobility, radiographic bone loss, pocket depth, and exudate were examined [9]. The eight vectors of the diagram in the IDRA include an assessment of a history of periodontitis, the percentage of sites with bleeding on probing, the number of teeth or implants with probing depths  ≥ 5 mm, the ratio of periodontal bone loss (evaluated from a radiograph) divided by the patient’s age, periodontitis susceptibility as described from the 2017 World Workshop on the Classification of Periodontal and Peri-implant Diseases [22] the frequency/compliance with supportive periodontal therapy, the distance from the restorative margin of the implant-supported prosthesis to the marginal bone crest, and prosthesis-related factors, including cleanability and fit of the implant-supported prosthesis [17]. A single clinician (T.Ş.) conducted the measurements in one session.

### Inclusion and exclusion criteria

Inclusion Criteria:

- 38 to 62 years of age.
- Systemically healthy or with a controlled medical condition.
- At least six months and at most five years passed after a dental implant’s functional prosthetic loading and received one or more dental implants with a fixed prosthesis.

Exclusion Criteria:

- Presence of uncontrolled systemic disease.
- Bruxism.

### Statistical analysis

Within the parameters of the investigation, statistical analyses were conducted using Statistics*. Numbers and percentages are used to convey categorical data. The mean and standard deviation are used to represent open-ended data. Two hundred ninety-eight dental implants in 80 patients were assessed within the study’s parameters. In the two groups who participated in the study, each group had 149 implants examined. To look into any discrepancies between the two groups investigated open-ended values, an independent sample t-test was employed. Categorical variables were compared using chi-square analysis. In this investigation, p < 0.05 was used as the statistical significance threshold.

## Results

Regarding age, sex, or implant duration, there were no statistically significant differences between smokers and nonsmokers (p > 0.05) (Table 1).

**Table 1:** Demographics of the study participants

		Smokers	Nonsmokers	P
**Age**		51.79 ± 10.41	49.39 ± 11.17	0.331
**Gender**	Male	22 (64.7%)	23 (50.0%)	0.139
	Female	12 (35.3%)	23 (50.0%)	

There is a statistically significant difference in the disease/health status evaluation of the examined implants of smoker and non-smoker participants (p < 0.05) (Table 2).

**Table 2:** Peri-implant health and occurrence of disease in patients

			Group		Total	p
			Smokers	Nonsmokers		
**Disease/Health**	**Peri-implant Mucositis**	n	37	59	96	0.001*
		%	24.8%	39.6%	32.2%	
	**Peri-implant Health**	n	100	64	164	
		%	67.1%	43.0%	55.0%	
	**Peri-implantitis**	n	12	26	38	
		%	8.1%	17.4%	12.8%	
**Total**		n	149	149	298	
		%	100.0%	100.0%	100.0%	

There was a statistically significant difference in the IDRA scores for the examined implants of smokers and nonsmokers (p < 0.05) (Table 3). The peri-implantitis group contained most patients with high IDRA scores; patient distributions for the other two disease categories differed. High IDRA scores were linked to failed implants in both groups (Table 4).

**Table 3:** Dental implant assessment tools and scales

		Smokers	Nonsmokers	p
**Dental Implant Health Scale**	1	141 (94.6%)	149 (100.0%)	0.042*
	2	3 (2.0%)	0 (0.0%)	
	3	3 (2.0%)	0 (0.0%)	
	4	2 (1.3%)	0 (0.0%)	
**Pink Esthetic Score**	0–9	79 (53.0%)	65 (43.6%)	0.114
	10	18 (12.1%)	25 (16.8%)	
	11–12	42 (28.2%)	39 (26.2%)	
	13–14	10 (6.7%)	20 (13.4%)	
**Keratinized Tissue Thickness**	1 mm and below	80 (53.7%)	66 (44.3%)	0.077
	2 mm	50 (33.6%)	69 (46.3%)	
	2 mm and above	19 (12.8%)	14 (9.4%)	
**Keratinized Tissue Width**	2 mm below	67 (45.0%)	55 (36.9%)	0.195
	2 mm and above	82 (55.0%)	94 (63.1%)	
**Vestibule Depth**	4 mm below	36 (24.2%)	36 (24.2%)	0.999
	4 mm and above	113 (75.8%)	113 (75.8%)	
**IDRA**	Low	10 (6.7%)	25 (16.8%)	
	Moderate	49 (32.9%)	21 (14.1%)	0.001*
	High	90 (60.4%)	103 (69.1%)	

There was no statistically significant difference between smokers and non-smokers regarding the pink esthetic scores of the implants under examination (p > 0.05) (Table 3). Whereas 69.8% of the individuals who did not smoke had pink esthetic ratings of 8 or higher, 30.2% of the participants who smoked had implants that were inspected, and 27.5% of the people who did not smoke had implants that were examined and had pink esthetic scores of less than 8. By contrast, the nonsmoking individuals’ implants had a pink esthetic score of 8 or above in 72.5% of the examined cases.

The implants’ keratinized gingival thickness and vestibule depth under examination showed no statistically significant differences between smokers and nonsmokers (p > 0.05). 53.7% of the examined implants of smokers were 2 mm and below, and 44.3% of the examined implants of non-smoking participants were 1 mm and down (Table 3).

There was a statistically significant difference in the dental implant health scale score for the examined implants of smokers and nonsmokers (p < 0.05). The non-smoking group had a complete implant health scale 1 (Table 3). The peri-implantitis group had fewer people with successful dental implants than the other groups (Table 4).

The peri-implantitis group had fewer people with successful dental implants than the other groups (p < 0.05) (Table 3).

**Table 4:** Dental implant health scale and distribution of IDRA scores in disease and health

		Peri-implant Mucositis	Peri-implant Health	Peri-implantitis	p
**Dental Implant Health Scale**	Success	95 (100.0%)	162 (98.8%)	33 (84.6%)	0.001*
	Survival	-	2 (1.2%)	4 (10.3%)	
	Failure	-	-	2 (5.1%)	
**IDRA**	Low	15 (15.6%)	18 (11.0%)	2 (5.3%)	0.003*
	Moderate	16 (16.7%)	46 (28.0%)	8 (21.1%)	
	High	65 (67.7%)	100 (61.0%)	28 (73.7%)	

## Discussion

IDRA is a risk assessment approach considering several characteristics that may help identify individuals at risk of getting peri-implantitis. According to the IDRA data, 56.3% of the 79 patients had a high risk, and 42.5% had a moderate risk. According to IDRA, peri-implantitis was found in 4 patients (12%) at moderate risk and 12 patients (27%) at high risk. Peri-implantitis is a common condition in patients with a high IDRA risk. [18]. De Ry et al. reported that increased IDRA was present in 28% of individuals with peri-implantitis. Fifteen implants at low risk had peri-implant mucositis (25%), and six implants at moderate risk had peri-implantitis (75%) [19]. Individuals with high-risk IDRA profiles seem to be more vulnerable to developing biological problems and implant loss [23]. In the present study, the two failing implants had high IDRA levels. Smokers had a greater frequency of high values. In the group with peri-implantitis, most implants showed high IDRA levels.

Peri-implant soft tissue position, texture, color, contour, and alveolar process deficiency were also measured and compared with a natural tooth reference (adjacent or contralateral to the study site) [24]. The thresholds for clinical acceptance are currently set at values of 8 to 14 for the PES [25]. It has been proposed that PES levels between 10 and 12 indicate good esthetic outcomes and that PES values between 13 and 14 indicate ideal implant esthetics [24]. In one study, all 45 anterior maxillary single-tooth implants examined fulfilled strict criteria for the success of implants concerning osseointegration; these criteria included the absence of peri-implant radiolucency, implant mobility, suppuration, and pain. The mean total PES of 7.8 ± 0.88 indicated favorable overall peri-implant soft tissue conditions [26]. PES values varied from 3.17 to 7.46 in a study evaluating 41 anterior implants [27]. After a year, the PES score for all 39 patients was 7.07 [28]. The average was 9.09 for smokers and 9.07 for nonsmokers in this research. In thirty single implants, the implant-related mean PES varied from 2.28 to 13.8 for single-tooth implants [15]. The pink esthetic score in the study ranged from 3 to 14.

The “success” category describes optimum conditions, the “survival” category describes implants that are functional but not associated with ideal conditions, and the “failure” of an implant indicates that the implant should be removed or has already been removed [9]. Based on a study’s measure for evaluating dental implant health, 98.4% were effective and in excellent condition [11]. In another study, the cumulative implant survival rate was 90.9% [23]. The dental implant health scale was higher in smokers than in nonsmokers. The success and survival rates on the implant health scale were reported to be 98.6% in smokers compared to 100% in nonsmokers.

Adequate peri-implant gingival height, a thick tissue phenotype, and a wide area of immobile keratinized gingiva may all help lower the risk of tissue inflammation and subsequent problems [29]. Compared to implant sites with ≥ 2 mm of keratinized gingiva, implant sites with a band of keratinized gingiva that was less than 2 mm in width were found to be more likely to experience pain when brushing, plaque accumulation, peri-implant soft tissue inflammation, greater probing depth, gingival recession, bleeding on probing, and peri-implantitis [30–32]. According to the findings of one study, smoking did not affect keratinized gingival width [33]. In another research, smokers’ keratinized gingival width was much more significant than that of nonsmokers [34]. The keratinized gingival width was significantly wider in nonsmokers in the current study.

The gingival biotype was considered thin if the measurement was ≤ 1.0 mm and thick if it was > 1.0 mm [10]. Soft tissues at the level of the supracrestal portion require a thickness of at least 2 mm to prevent the occurrence of mucosal recession and unesthetic tissue discoloration [35]. When the mucosal tissue is 2 mm or less in width, significant crestal bone loss might be expected. The initial gingival tissue thickness at the crest may significantly influence marginal bone stability around implants [36]. If the tissue thickness is 2.0 mm or less, crestal bone loss of up to 1.45 mm may occur [37]. In the current study, 46.3% of the examined implants of the smoking participants were 2 mm or thicker, whereas 55.7% of the examined implants of the nonsmoker participants were.

The study confirmed that shallow vestibule depth is associated with higher mucosal recession, a higher rate of relative attachment loss, radiographic bone level, greater bleeding on probing, gingival index, and peri-implant failure compared with sites at which there is adequate vestibule depth ( > 4 mm). Moreover, sites with shallow vestibule depth presented a lower keratinized mucosa width than sites with adequate vestibule depth [38]. The average implant circumference vestibule depth measurement in patients who smoke was 5.93 ± 2.95, while that in patients who do not smoke was 5.94 ± 2.70. While 55.0% of the implants of the smoking participants had mucosal tissue that was 2 mm or greater in width, 63.2% of the nonsmoking participants did.

## Conclusions

Smoking, with other risk factors, has a considerable impact on the success or failure of implant surgery, the individual’s risk status, and the width of keratinization. The results of this study suggest that there is a need to increase patient awareness regarding the benefits of smoking cessation or reduction and to promote such efforts actively. Additionally, it is essential to provide support to patients in their endeavors, considering the detrimental effects of smoking on individuals with implants.

## Data Availability

The datasets analyzed during the current study are available from the corresponding author upon reasonable request.

## Abbreviations

*IBM SPSS Version 26.0: IBM Corp., Armonk, NY, USA
IDRA: Implant Disease Risk Assesment
PES: Pink Esthetic Score
